# Metabolic and OXPHOS Activities Quantified by Temporal *ex vivo* Analysis Display Patient-Specific Metabolic Vulnerabilities in Human Breast Cancers

**DOI:** 10.3389/fonc.2020.01053

**Published:** 2020-06-30

**Authors:** Andre Koit, Natalja Timohhina, Laura Truu, Vladimir Chekulayev, Shivakumar Gudlawar, Igor Shevchuk, Katrin Lepik, Lea Mallo, Riina Kutner, Vahur Valvere, Tuuli Kaambre

**Affiliations:** ^1^Chemical Biology Laboratory, National Institute of Chemical Physics and Biophysics, Tallinn, Estonia; ^2^Oncology and Hematology Clinic at the North Estonia Medical Centre, Tallinn, Estonia; ^3^School of Natural Sciences and Health, Tallinn University, Tallinn, Estonia

**Keywords:** mitochondrial flux, ADP-activated respiration, OXPHOS, citrate, glutaminolysis, metabolic dependencies, cancer metabolism, predictive biomarker

## Abstract

Research on mitochondrial metabolism and respiration are rapidly developing areas, however, efficient and widely accepted methods for studying these in solid tumors are still missing. Here, we developed a new method without isotope tracing to quantitate time dependent mitochondrial citrate efflux in cell lines and human breast cancer samples. In addition, we studied ADP-activated respiration in both of the sample types using selective permeabilization and showed that metabolic activity and respiration are not equally linked. Three times lower amount of mitochondria in scarcely respiring MDA-MB-231 cells convert pyruvate and glutamate into citrate efflux at 20% higher rate than highly respiring MCF-7 mitochondria do. Surprisingly, analysis of 59 human breast cancers revealed the opposite in clinical samples as aggressive breast cancer subtypes, in comparison to less aggressive subtypes, presented with both higher mitochondrial citrate efflux and higher respiration rate. Additionally, comparison of substrate preference (pyruvate or glutamate) for both mitochondrial citrate efflux and respiration in triple negative breast cancers revealed probable causes for high glutamine dependence in this subtype and reasons why some of these tumors are able to overcome glutaminase inhibition. Our research concludes that the two widely used breast cancer cell lines fail to replicate mitochondrial function as seen in respective human samples. And finally, the easy method described here, where time dependent small molecule metabolism and ADP-activated respiration in solid human cancers are analyzed together, can increase success of translational research and ultimately benefit patients with cancer.

## Introduction

Research in cancer metabolism is expanding despite seminal discoveries were made almost a century ago, when the Warburg effect was first described by three metabolic properties—elevated glucose uptake with increased lactate secretion despite the presence of oxygen (1). Decades of research has been conducted, but the nature of this metabolic phenotype is still poorly understood (2), however, it has become common knowledge, in contrast to Warburg's initial hypotheses, that mitochondria in cancer cells remain functional (3–5).

In malignancies, mitochondria have become metabolic hubs, that feed numerous cytosolic processes warranting cell growth and disease progression (6, 7) with two major extracellular sources for mitochondrial function being glucose and glutamine (6, 8). Despite cancers are able to direct glucose in the form of pyruvate into mitochondria and conduct oxidative phosphorylation (OXPHOS), part of the canonical tricarboxylic acid cycle (TCA) required for OXPHOS is truncated and citrate is directed out of the mitochondria (9). Mitochondrial citrate efflux can be further increased by reductive carboxylation of glutamate, which reverses the traditionally known direction of TCA (10) and emphasizes importance of metabolic flexibility in cancers. Citrate is cleaved in the cytosol by ATP citrate lyase, and the products are used for *de novo* lipid synthesis (9) or feed into downstream pathways necessary for synthesis of cholesterol, isoprenoids or protein acetylation (11). Among others, ATP citrate lyase and glutamine utilization have been considered as potential antineoplastic targets and even though these processes are active in most cancers, biomarkers for selecting suitable patients for respective treatments have remained unknown.

Isotope tracing has been widely used to trace nutrient faith in metabolic pathways and this method has led to numerous discoveries (10, 12). Isotope tracing is mainly used on cultured cells, but similar analysis has been optimized also for human cancer patients. In humans, this requires intravenous preoperative administration of labeled metabolite, rapid freezing of the target samples after resection and other non-standard interventions during surgery (4), which in turn can complicate wider use of this method. Despite the rapid nature of the metabolic experiments on human cancers, solute transporters and enzymes can have residual activity even at very low temperatures and can severely affect the results (13). Shortcomings in metabolomic studies have been noted by numerous groups and many of them have developed new methods to differentiate mitochondrial and cytosolic metabolite pools or metabolite fluxes within cells to answer critical questions in understanding cancer metabolism (14–17). Reliable, efficient and widely accepted methods for studying metabolism in solid tumors, however, are still missing.

Mitochondrial function is central in understanding metabolic activity of cancers cells, but interestingly OXPHOS, as a central aspect of mitochondrial functionality, has received only very limited attention by researchers. Functional studies on respiration using selective permeabilization of cells' outer membrane have long been conducted in cardiac and skeletal muscle samples (18, 19), but in solid human tumors, the research on OXPHOS is scarce and used by very limited number of groups (5, 20, 21). Selective permeabilization removes cholesterol from the outer membrane of cells and equalizes the cytosolic compartment with the reaction medium while mitochondria in the samples remain fully functional, maintain functional connections to cytoskeletal structures and can be directly manipulated with exogenous substances to expand knowledge on mitochondrial metabolism (19, 20, 22). However, OXPHOS studies on solid human cancer samples are preferably conducted by laboratories having experience in applying permeabilization method on healthy tissues (like muscles), but that in turn limits availability of this technique outside specialized centers.

In the present work, we study respiration in breast cancer cell lines and human breast cancer samples and simultaneously present and use newly developed metabolomic method. By combining these two approaches, we describe breast cancer mitochondrial function in time dependent manner and bring out dependencies not evident by using either approach alone. Importantly, human breast cancer samples are used in addition to cell lines as translational research needs better ways to understand subcellular metabolomic processes in order to help patients with this devastating disease.

## Materials and Methods

### Chemicals

All chemicals were purchased from Sigma-Aldrich Chemical Com. (USA) and were of the highest purity available (>98%).

### Clinical Samples and Medical Data

The tissue samples were provided by the Oncology and Hematology Clinic at the North Estonian Medical Centre (Tallinn). All the samples were analyzed within 6 h after surgery. Only primary tumors were examined and information from respective pathology reports was provided by the North Estonian Medical Centre for all the analyzed samples. Informed consent was obtained from all the patients and coded identity protection was applied. All investigations were approved by the Tallinn Medical Research Ethics Committee and were in accordance with Helsinki Declaration and Convention of the Council of Europe on Human Rights and Biomedicine. The entire group consisted of 59 patients with breast cancer (57 females, 2 males).

### Cell Cultures

MDA-MB-231 and MCF-7 cells were grown as adherent monolayers in low glucose (1.0 g/L) or high glucose (5 g/L) Dulbecco's Modified Eagle's Medium (DMEM) with stable L-glutamine (2 mM) and sodium pyruvate (from CAPRICORN scientific) supplemented with 10% heat-inactivated fetal bovine serum, 10 μg/mL human recombinant Zn-insulin, and antibiotics: penicillin (100 U/ml), streptomycin (100 μg/ml) and gentamicin at a final concentration of 50 μg/ml. Cells were grown at 37°C in humidified incubator containing 5% CO_2_ in air and were sub-cultured at 90% confluence.

### Mitochondrial Respiration in Saponin-Permeabilized Tissue and Cell Culture Samples

Analysis was conducted as described previously (5). In brief—respiratory activity of tumor and control tissues *in situ* was captured using skinned sample technique (18, 20, 23, 24). This method allows analysis of the function of mitochondria in their natural environment and leaves links between cytoskeletal structures and mitochondrial outer membrane intact (22, 25–27). For cell lines, direct permeabilization in the oxygraphic chambers was used (21). Cytochrome c test was used to confirm integrity of the mitochondrial outer membrane (20, 23, 24, 28); mitochondrial inner membrane quality was checked using carboxyatractyloside test as the last procedure in every experiment (20, 23, 24, 28, 29). Rates of O_2_ consumption were assayed at 25°C using Oxygraph-2k high-resolution respirometer (Oroboros Instruments, Innsbruck, Austria) loaded with pre-equilibrated respiration buffer—medium-B (20). All rates of respiration (V) are expressed in nmol O_2_/min per mg wet tissue weight for solid tumors and in nmol O_2_/min per million cells for cell cultures.

### Mitochondrial Metabolite Efflux in Saponin-Permeabilized Samples

Experiment setup was equal to that described in paragraph 2.4. Oxygraph-2k respirometer chamber was loaded with pre-equilibrated respiration buffer and a single type of selectively permeabilized sample was divided into two oxygraphy chambers. Intended substrates (pyruvate or glutamate; both additionally supplemented with 2 mM malate) were injected and state 2 respiration was captured (30). In one of the parallel chambers, the sample was incubated without exogenous inhibitors, but in the other chamber mitochondrial citrate efflux was inhibited by addition of 1,2,3-benzene tricarboxylic acid (BTC) that selectively halts the function of mitochondrial citrate transporter (31). This parallel setup allows to differentiate citrate generated by mitochondria and citrate possibly generated by leftover cytosolic processes. Reaction was initiated by addition of 2 mM ADP.

Samples collected at different time points were immediately frozen in liquid nitrogen or cooled on ice-bath. Analysis of the samples was done using UV-VIS spectroscopy as described elsewhere (32) or GC-MS. Citrate content in BTC-supplemented parallel was termed to be extramitochondrial.

In the cytosol, ATP-citrate lyase can turn citrate into oxaloacetate and acetyl-CoA (11), and hence affect outcome of the analysis. To eliminate this reaction and allow uninterrupted accumulation of citrate produced by mitochondria, an inhibitor for ATP-citrate lyase was used in all experiments (BMS-303141). Suitability of the described method was further confirmed by running the analysis on cell lines without permeabilization, without addition of ADP or addition of ADP at 1 or 2 mM levels and concentration of BTC was titrated to best suit human breast cancer samples (data not shown).

### Analysis of Metabolites

For GC-MS analysis on cell lines, the cells were allowed to grow to 90% confluence on d35 dishes. For collection, the dishes were placed on ice and growth medium aspirated. One milliliter of extraction solvent (80% methanol in water; stored at −80°C at least 1 h before use) was added and the dish was stored at −80°C for 15 min. The cell layer was thereafter scraped from the dish and the mixture was centrifuged at 20,000 g/4°C for 15 min. Supernatant was divided into two and the pellet was analyzed for protein content. In preparation of derivatization process, the supernatant was freeze-tried. For derivatization, 20 mL 20 mg/ml O-methoxylamine in pyridine solution was added and the mixture incubated in heater at 30°C for 90 min. Thereafter 80 mL of MSTFA/1%TMCS was added and the mixture was incubated in heater at 37°C for 30 min. Derivatized sample was centrifuged at 15,000 g for 15 min and supernatant transferred to 200 mL vial inserts placed in 2 mL vials for analysis on GC-MS.

The GC-MS analysis was performed on an Agilent GCMS MSD. Five microliters of each sample was injected with split less injection mode, with 120 s purge time at 9.4 psi and 3.1 ml/min helium purge flow, helium column carrier gas flow was maintained at 1 ml/min and flow rate of 20 ml/min for 3 min to purge the injector. Ion source temperature and quadrupole temperature were maintained at 230 and 150°C.

Chromatographic separation was performed using HP-5% phenylmethyl siloxane −30^*^250^*^0.25 μm. The front inlet temperature was maintained at 250°C and helium was used as carrier gas. Column temperature was maintained at 60°C for 1 min and then held for 1 more min, then increased to 325°C with 10° per minute and held for 10 min followed by post-runtime of 5 min at 60°C with a total runtime of 42.5 min. Solvent delay was set at 5.9 after making sure that pyridine, MSTFA peaks are non-detectable and pyruvate and lactate peaks are detectable.

### Western Blot Analysis of the Level of Citrate Synthase Expression

Post-operative tissue samples (70–100 mg) were crushed in liquid nitrogen and homogenized in 20 volumes of RIPA lysis buffer [50 mM Tris-HCl pH 8.0, 150 mM NaCl, 2 mM EDTA, 0.5% sodium deoxycholate, 0.1% SDS, 0.1% Triton X-100, and complete protease inhibitor cocktail (Roche)] by Retsch Mixer Mill at 25 Hz for 2 min. After homogenization, samples were incubated for 30 min on ice and centrifuged at 12,000 rpm for 20 min at 4°C. The proteins in the supernatants were precipitated using acetone/TCA to remove non-protein contaminants. Briefly, supernatants were mixed with 8 volumes of ice-cold acetone and 1 volume of 100% TCA, kept at −20°C for 1 h and then pelleted at 11,500 rpm for 15 min at 4°C. The pellets were washed twice with acetone and resuspended in 1× Laemmli sample buffer. Proteins were separated by polyacrylamide gel electrophoresis, transferred to a polyvinylidene difluoride (PVDF) membrane and subjected to immunoblotting with anti-citrate synthase antibody (Abcam, ab96600). Then, the membranes were incubated with corresponding horseradish peroxidase-conjugated secondary antibody and visualized using an enhanced chemiluminescence system (ECL; Pierce, Thermo Scientific). After chemoluminescence reaction, the PVDF membranes were stained with Coomassie brilliant blue R250 to measure the total protein amount. The signal intensities were calculated by ImageJ software and normalized to total protein intensities.

### Citrate Synthase Activity

Citrate synthase activity was measured in whole-cell extracts at 412 nm using 5,5′-dithio-bis(2-nitrobenzoic acid) as described elsewhere (33).

### Confocal Microscopy Imaging

Cells were seeded on glass coverslips in 12-well plates (Greiner bio-one) and allowed to adhere overnight, then passaged onto Matrigel coated glass coverslips and cultured in 12-well plates for 3 days. Then, the growth medium was removed, and cell samples treated *via* selective marking of mitochondrial outer membrane translocase Tom20 (Santa Cruz Biotechnology, sc17764). The Tom20 fluorescence intensity was normalized against whole β-tubulin (Abcam®, ab6046) fluorescence. After incubation, cells were washed with PBS and incubated with corresponding fluorescence-conjugated secondary antibodies. Finally, cells were incubated for 15 min with 4′,6-diamidino-2-phenylindole dihydrochloride (DAPI, Molecular Probes™) to visualize the cell nucleus. Cells were imaged by an Olympus FluoView FV10i-W inverted laser scanning confocal microscope.

### Data Analysis

Data in the text, tables, and figures are presented as mean ± standard error (SEM). Results were analyzed using analysis of variance (ANOVA) and Student's *t*-test, *p*-values <0.05 were considered statistically significant.

## Results

**Figure 1:**
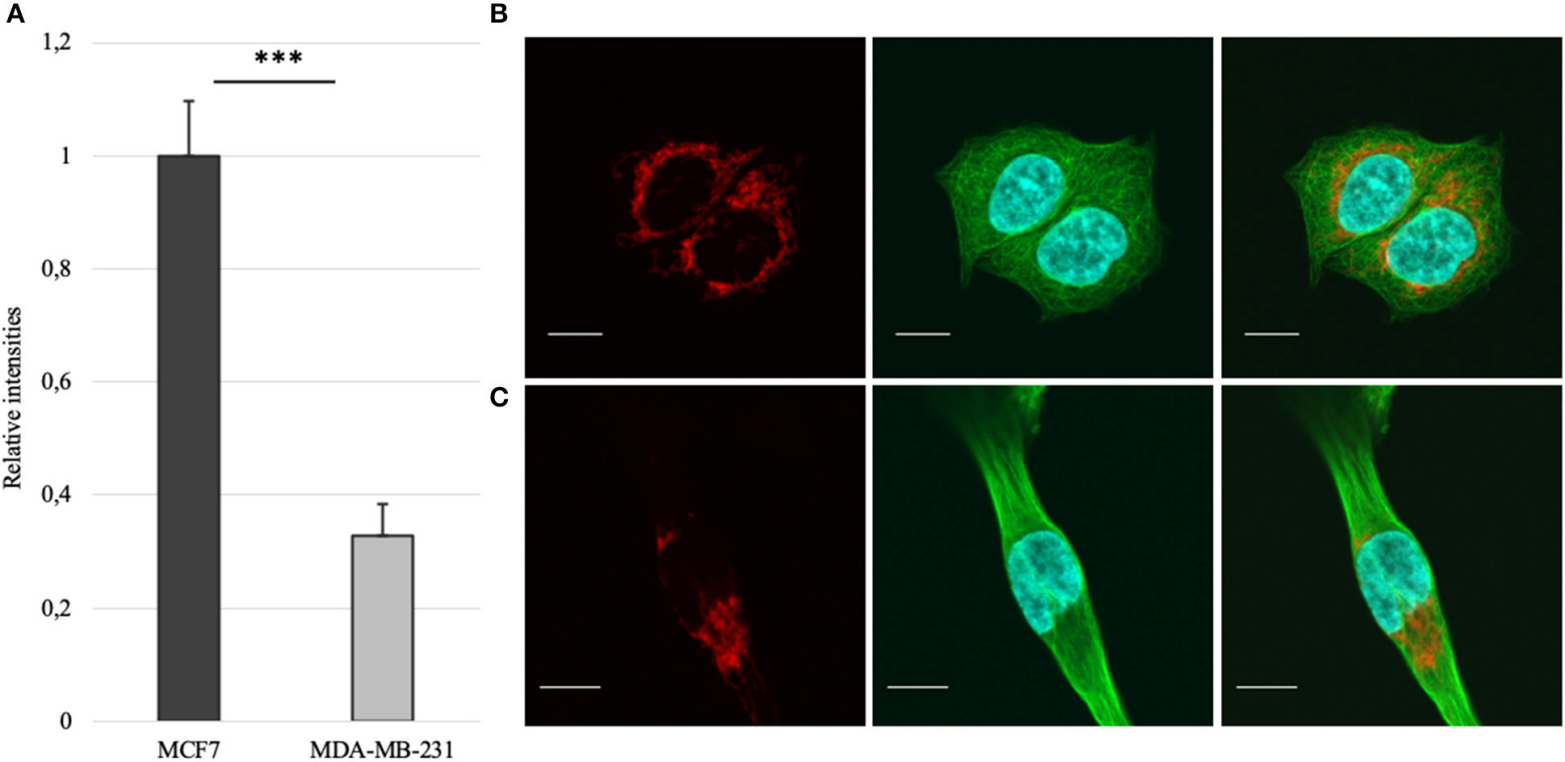
**(A)** Mitochondrial amount in MCF-7 vs. MDA-MB-231 cells as assessed from confocal imaging [staining of mitochondria (red), whole β-tubulin (green) and nucleus (blue) on figures **(B)** for MCF-7 and **(C)** for MDA-MB-231; white bar 10 μm]. ***p < 0.001.

**Figure 2:**
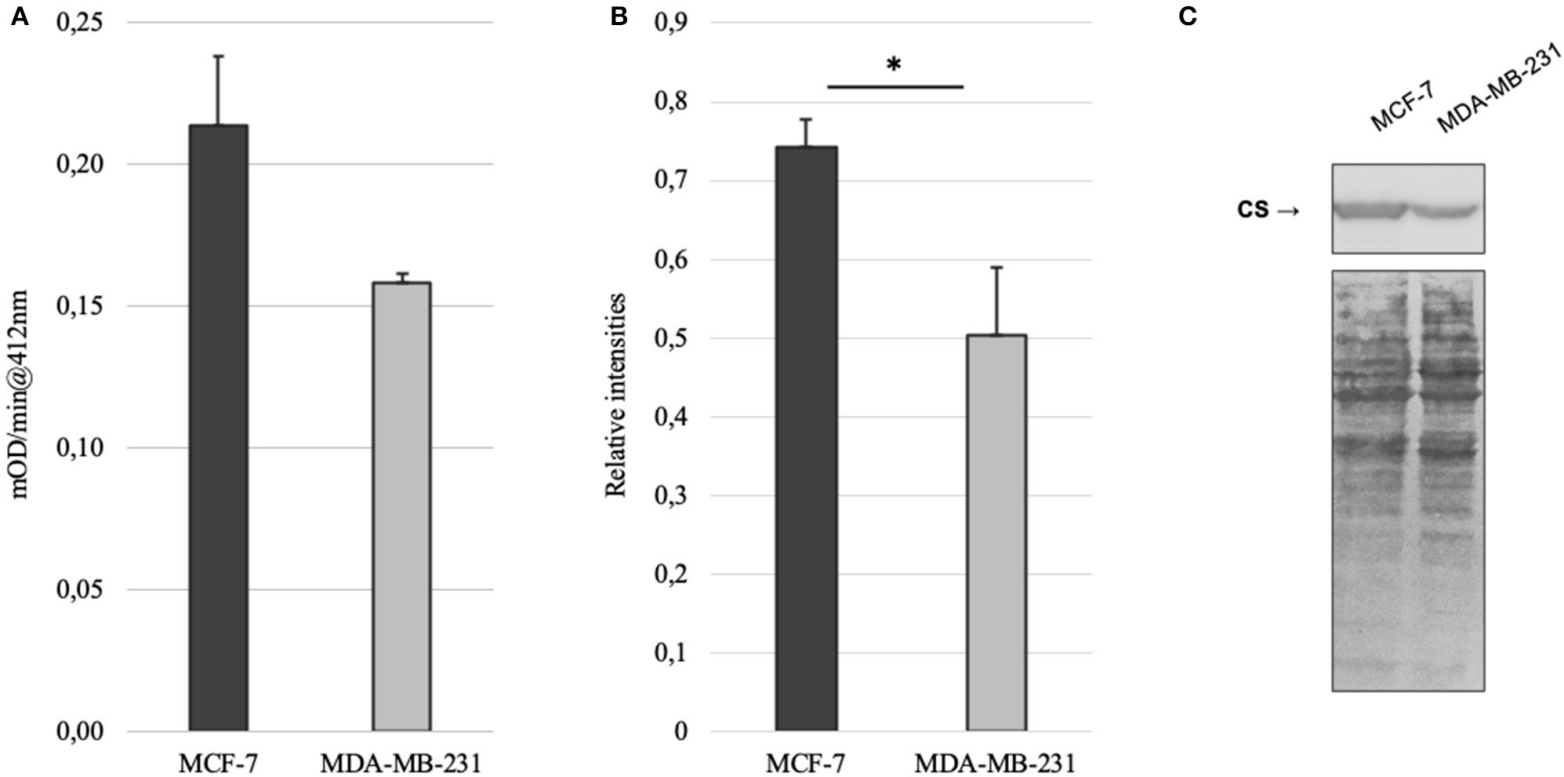
**(A)** Citrate synthase (CS) activity in MCF-7 and MDA-MB-231 cell lines; **(B)** relative expression of citrate synthase based on western blot imaging in MCF-7 and MDA-MB-231 cell lines; **(C)** western blot images for citrate synthase and whole protein in MCF-7 and MDA-MB-231 cell lines. *p < 0.05.

**Figure 3:**
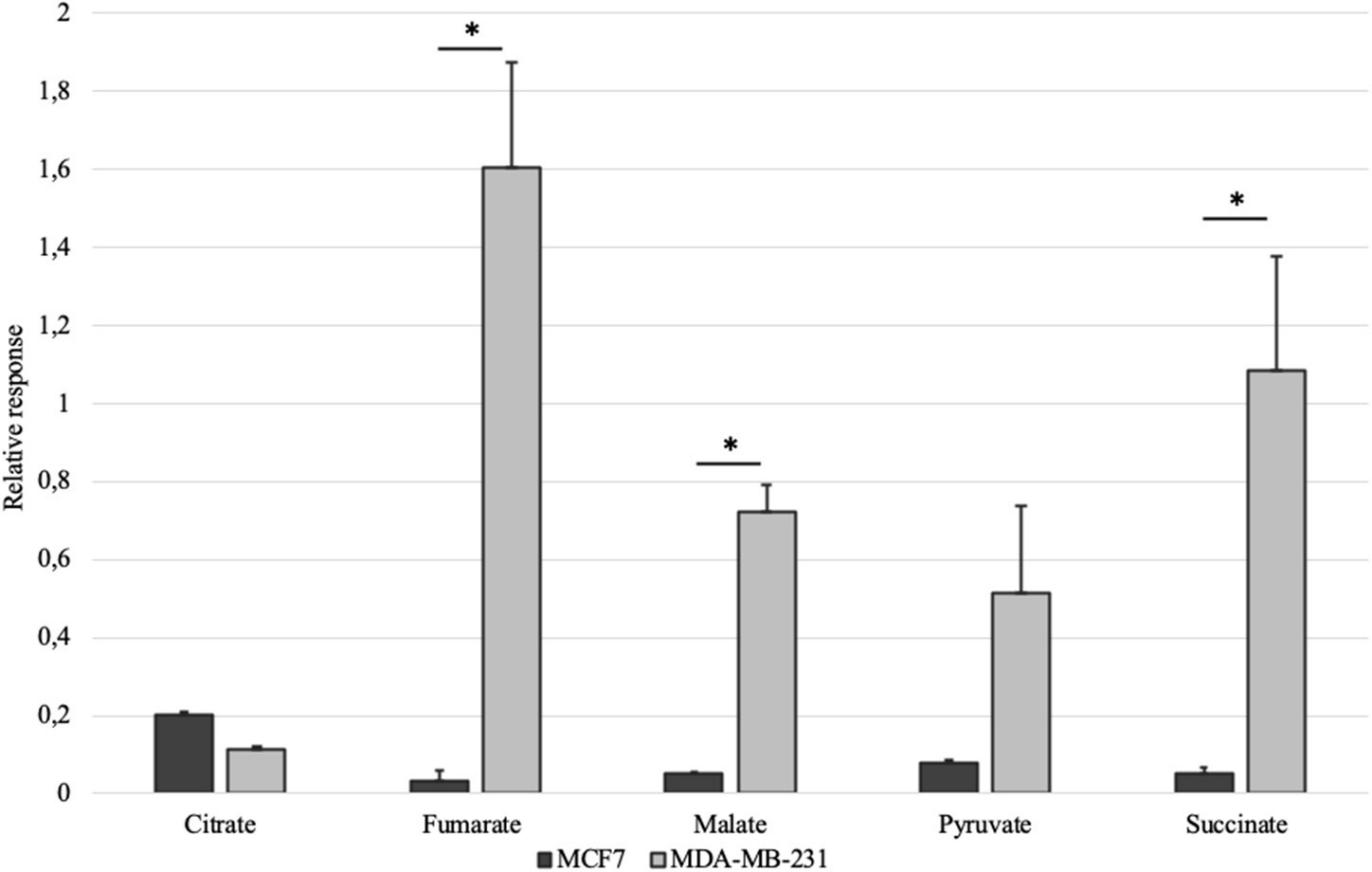
Selected citric acid cycle metabolites in whole-cell lysates of MCF-7 vs. MDA-MB-231 cells normalized to internal standard and protein content. *p < 0.05.

Mitochondrial amount in MCF-7 and MDA-MB-231 was examined by confocal microscopy and calculated using ImageJ software. Analysis revealed that mitochondrial amount in MCF-7 cells is about three times higher than that in the MDA-MB-231 cells and the difference was significant (Figure 1). Mitochondrial content was additionally analyzed using citrate synthase activity and even though difference in the results was not found to be significant, results were similar to that found via confocal microscopy (Figure 2). Citrate synthase was also studied on expression level using Western blot analysis and the results were confirming that there is more citrate synthase in MCF-7 cells (Figure 2). These findings were in good agreement with our previous research results where we showed using permeabilization that ADP-initiated respiration rate in MCF-7 cells is about 2.5–3 times higher than that in MDA-MB-231 cells (5). As we were interested in understanding links between mitochondrial respiration and mitochondrial metabolism, we thereafter investigated the levels of citric acid cycle metabolites in the two cell lines. GC-MS analysis revealed that relative citrate content in MCF7 and MDA-MB-231 is similar (content of citrate is about 2 times higher in MCF7 cells), but in opposite to other results described above, levels of all other studied metabolites were found to be significantly higher in the MDA-MB-231 cells (Figure 3).

The results for mitochondrial amount, ADP-activated respiration, whole cell citrate content, citrate synthase activity and abundance of the same enzyme showed similar results. However, very low level of citrate in MDA-MB-231 cells, in relation to other TCA metabolites in the same lysates, could not be easily explained. Based on these findings we decided to concentrate on citrate in the following study due to its numerous roles in maintaining cancer cell homeostasis and in connecting mitochondrial and cytosolic processes (11, 34, 35). We concluded that steady-state metabolite analysis as used above, is not sufficient in understanding the role of citrate and we need to measure TCA metabolic activity on functional level to see the process in time dependent manner. In order to achieve that, we designed a simple experiment to measure mitochondrial citrate efflux (MCE) in selectively permeabilized samples. Respirometry and metabolic methods were also incorporated into the new method as described in the Methods section.

**Figure 4:**
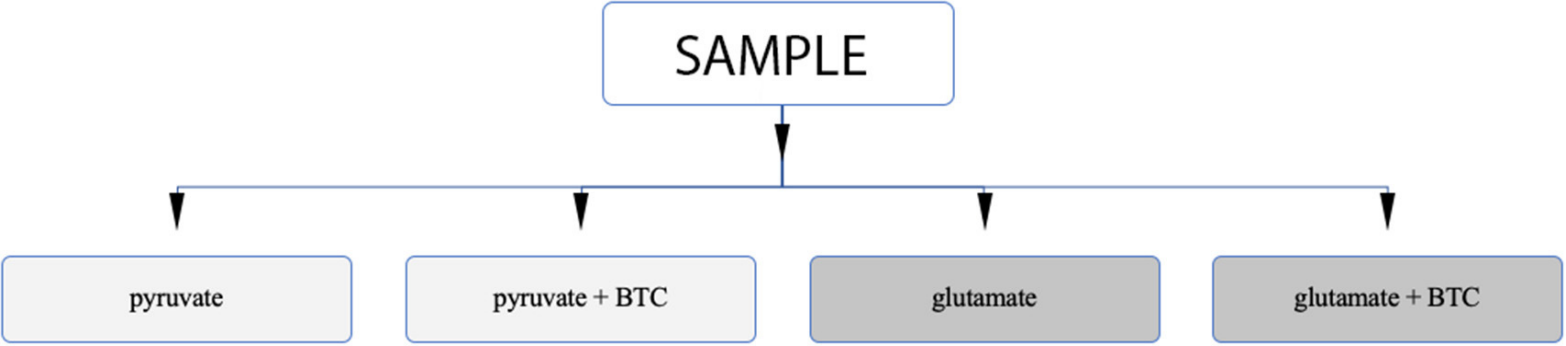
Analysis algorithm to differentiate if citrate produced by mitochondria in permeabilized samples is originating from pyruvate or glutamate as the main carbon source. One type of sample is analyzed under four different settings.

**Figure 5:**
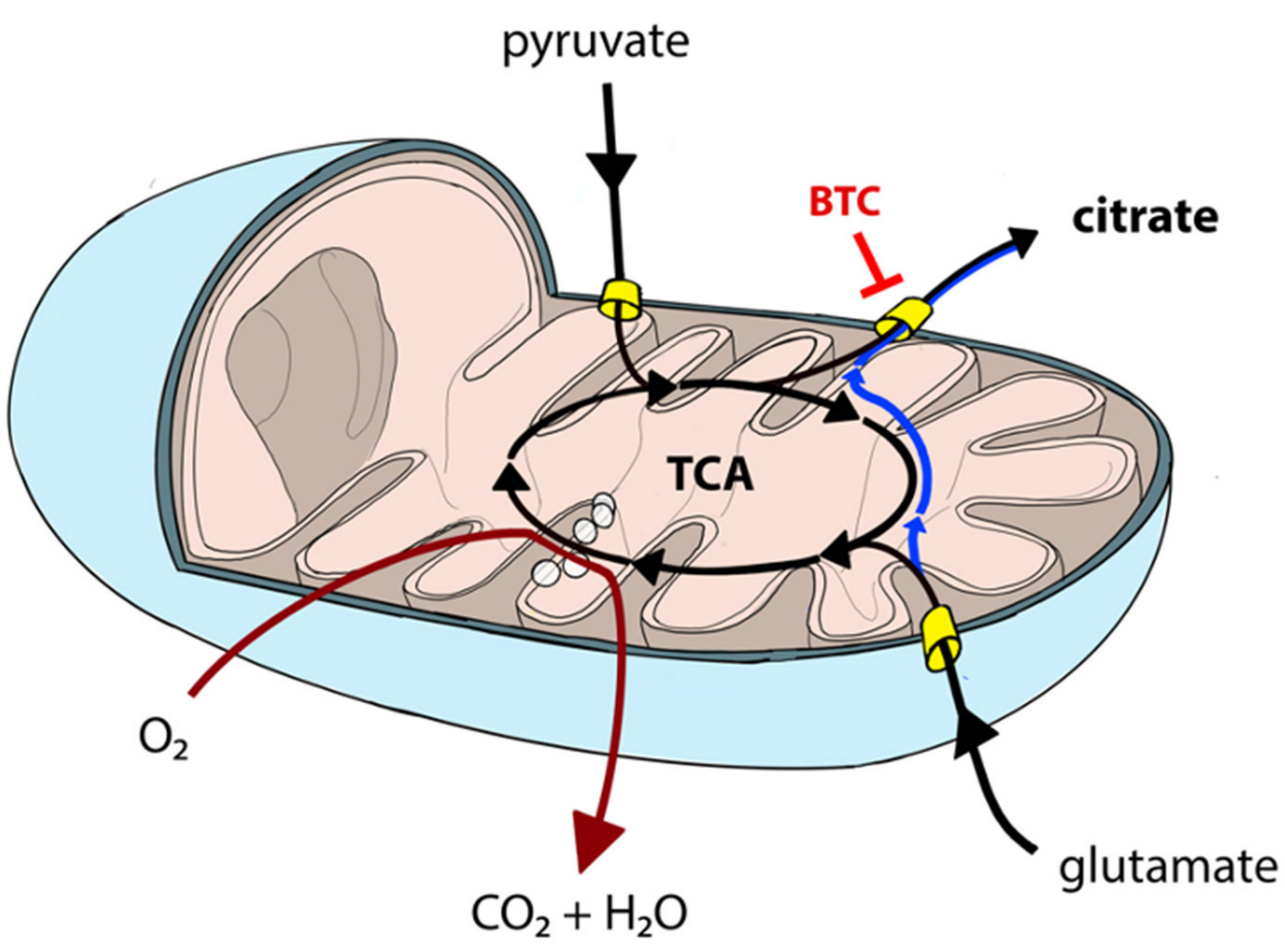
Canonical TCA in mitochondria can be truncated resulting in pyruvate-derived carbon being transported back to cytosol as citrate (black lines). Similarly, glutamate can be converted into mitochondrial citrate efflux via reductive carboxylation (blue line) or used in canonical TCA (black lines). Mitochondrial citrate efflux from both sources can be inhibited by BTC. TCA and OXPHOS (dark red line) processes are partially overlapping and, hence, need to be analyzed together to comprehensively describe mitochondrial function.

Permeabilization and use of Oroboros respirometer allows to manipulate availability of mitochondrial substrates that the sample can use. Using that option, we tested each sample separately with either pyruvate or glutamate as the main nutrient and both parallels were accompanied by additional sample supplemented with BTC (Figure 4). These two substrates were selected as mitochondrial citrate can mainly be synthesized through canonical TCA from pyruvate or through reductive carboxylation from glutamine derived glutamate (10) as depicted on Figure 5.

**Figure 6:**
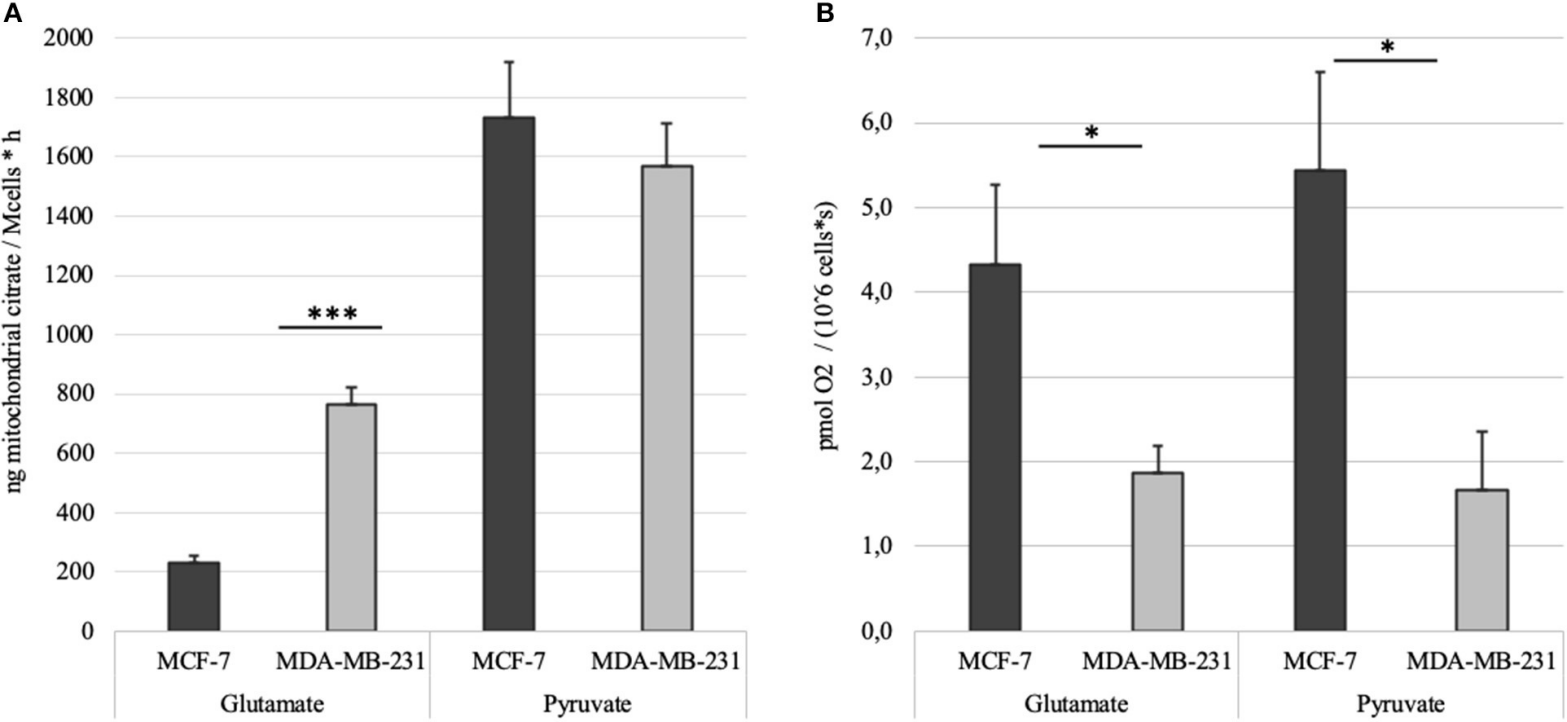
**(A)** Time dependent mitochondrial citrate efflux (MCE) in MCF-7 and MDA-MB-231 cell lines with either pyruvate or glutamate as the main nutrient. Results are corrected with citrate accumulation rates registered for parallel BTC tests. **(B)** ADP-activated respiration rates in MCF-7 and MDA-MB-231 cells measured during MCE experiments. *p < 0.05; ***p < 0.001.

MCE was measured in MCF-7 and MDA-MB-231 cell lines as described above. Citrate accumulation into the reaction medium was stopped at 60 min post-addition of 2 mM ADP. Net MCE was calculated by subtracting result registered in the BTC supplemented parallel from that without added BTC (Figure 6A). Tests revealed that mitochondria in both of the cell lines generate similar amount of citrate if pyruvate is used as the main carbon source. However, significant differences appear if glutamate is used. Specifically, mitochondria of MDA-MB-231 cells efflux citrate in the presence of glutamate at 3 times higher rate than MCF7 cells. In addition, if MCE rates from both pyruvate and glutamate are summarized, then MDA-MB-231 cells, in comparison to MCF-7 cells, are able to direct 20% more external carbon into cytosolic processes through citrate. We thereafter decided to measure MCE for clinical samples to confirm if differences are present between the used model systems and real samples. Experiment was set up equally to that used for cell lines.

**Figure 7:**
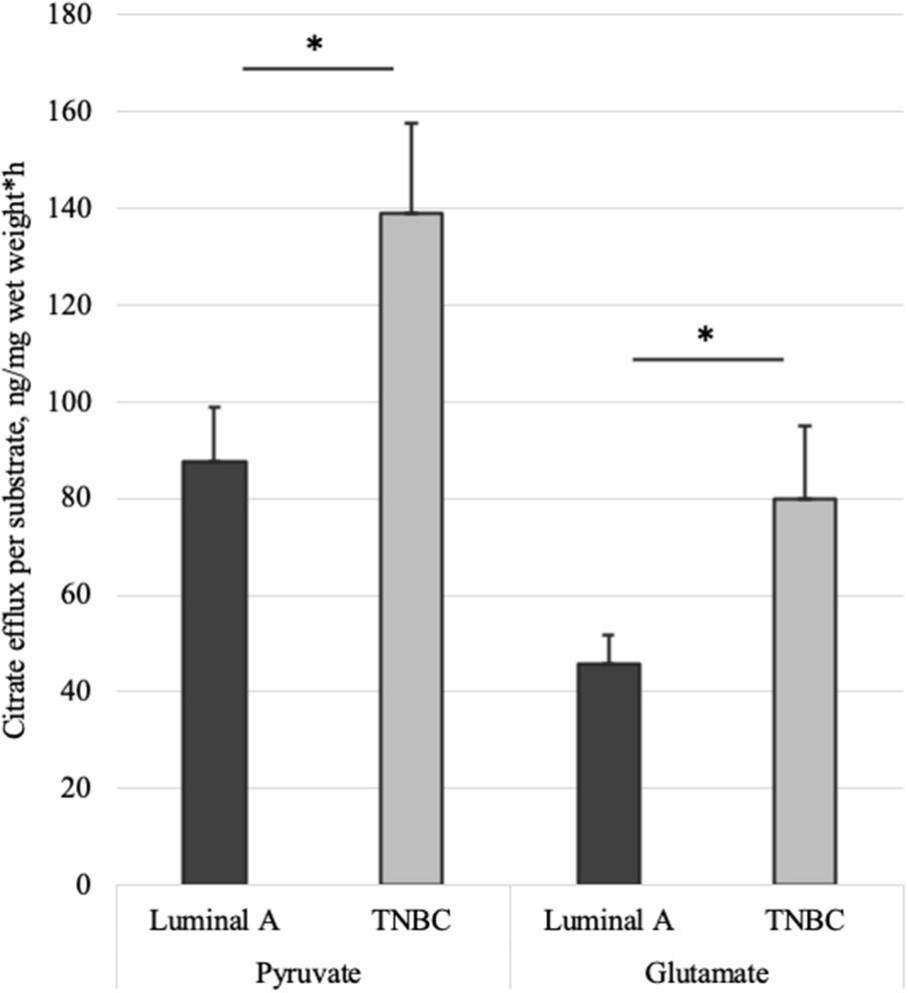
Mitochondrial citrate efflux (MCE) measured for pyruvate or glutamate as the metabolic substrate in Luminal A and TNBC human breast cancer samples. MCE measured as ng citrate/mg wet tumor weight*h; Luminal A 11 patients, TNBC 7 patients. *p < 0.05.

**Figure 8:**
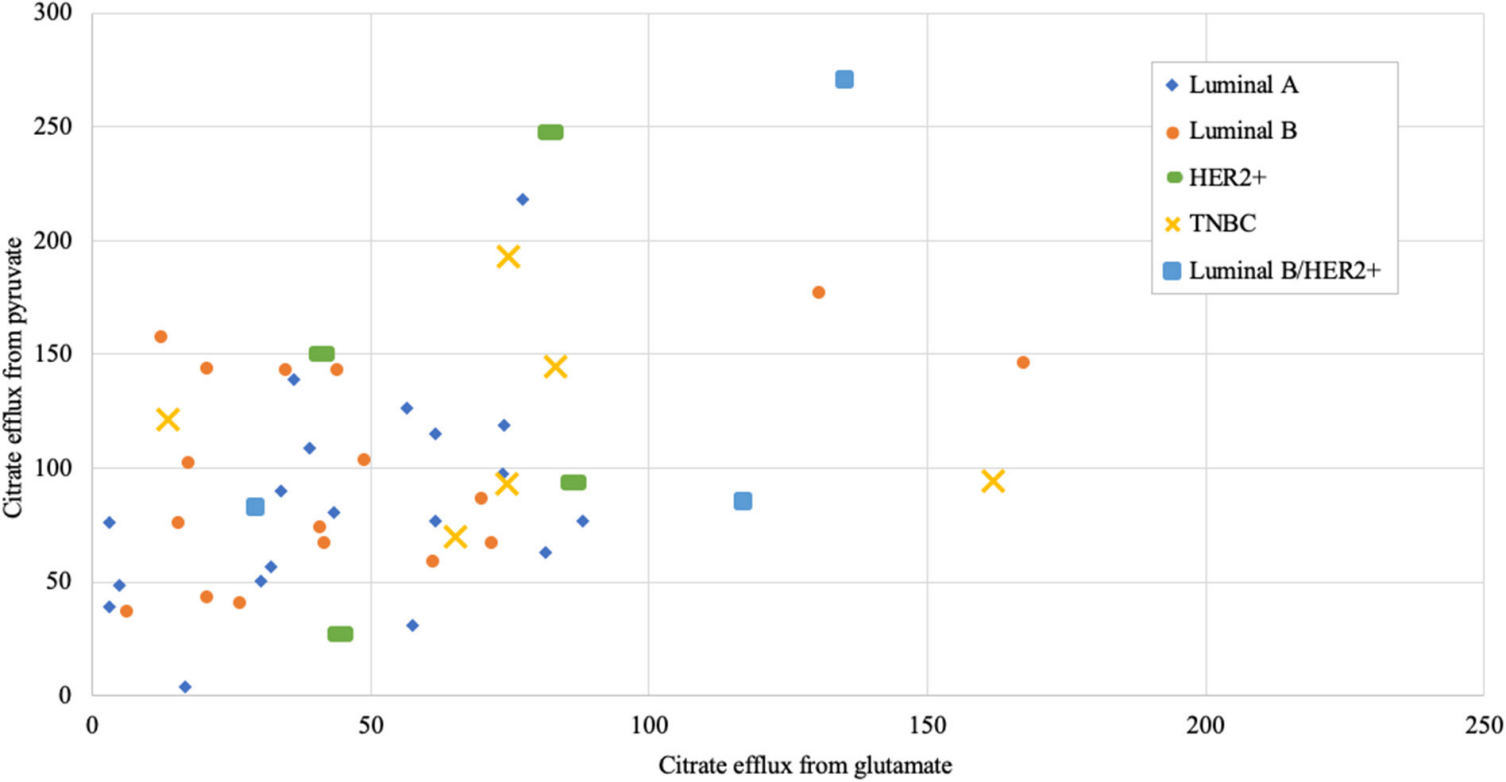
Mitochondrial citrate efflux (MCE) values from pyruvate vs. from glutamate as main metabolic substrate measured in human breast cancer samples (MCE measured as ng citrate/mg wet tumor weight*h; Luminal A 19, Luminal B 17, HER2+ 4, TNBC 6, Luminal b/HER2+ 3 patients).

**Figure 9:**
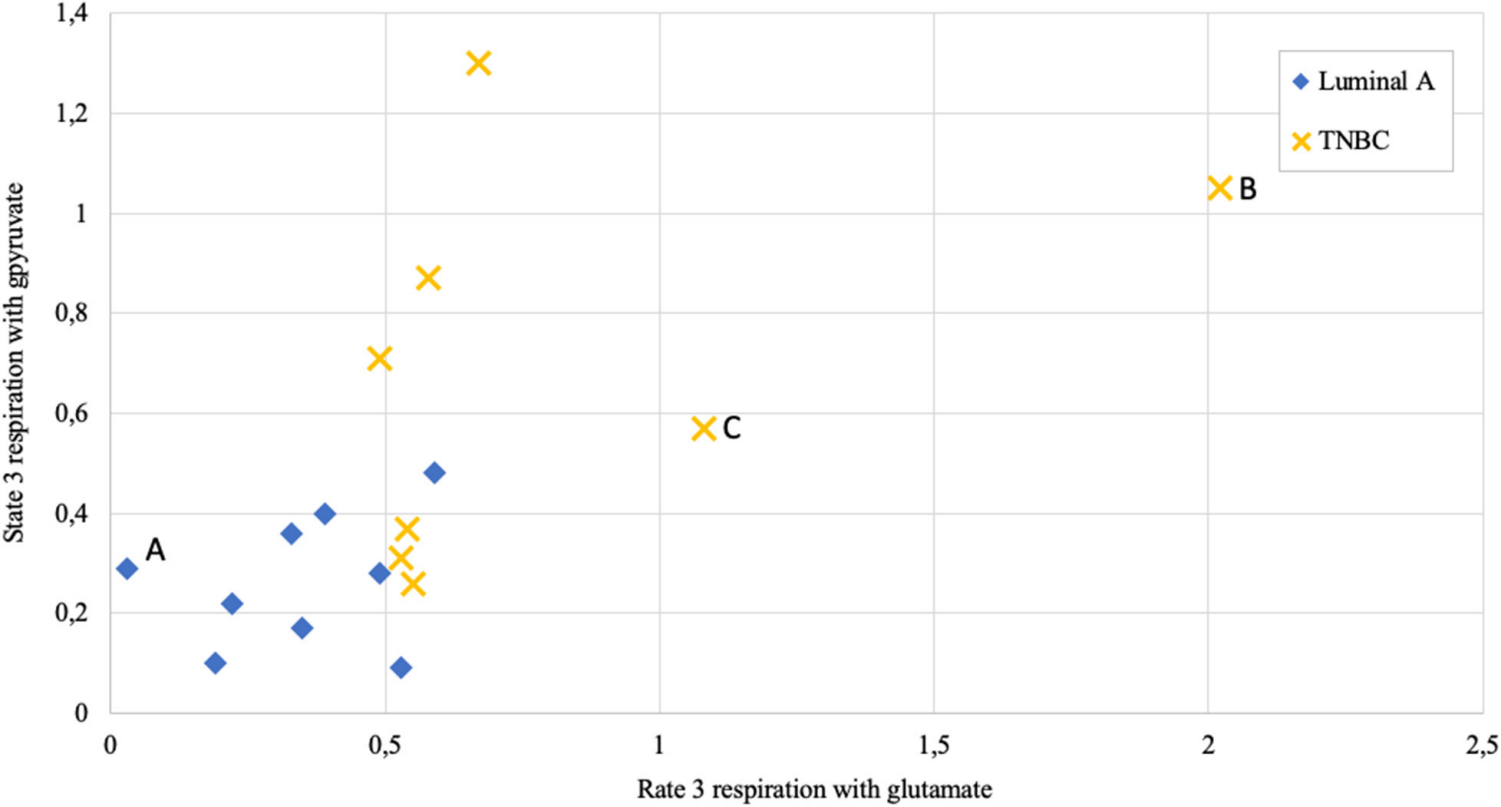
State 3 respiration rate dependence on metabolic substrate (pyruvate vs. glutamate) in human breast cancer samples; measured as nmol O_2_/min per mg wet; Luminal A 9 patients, triple negative breast cancer (TNBC) 8 patients. Letters A, B, and C as discussed in the text.

Altogether 59 human breast cancer samples were analyzed for both MCE and ADP-activated respiration in the presence of either pyruvate or glutamate as mitochondrial substrate. Quantity of tissue samples from five patients were not sufficient to apply the full testing algorithm (Figure 4). Every performed experiment, however, always had BTC-supplemented parallel done to differentiate mitochondrial citrate efflux from residual cytosolic activity. The MCE rates for all samples were grouped based on breast cancer molecular subtypes (Table 1) and results for the least aggressive (Luminal A) and most aggressive (triple negative—TNBC) were plotted (Figure 7), respectively to present significant differences found for those two clinically opposing subtypes. Finally, for all human samples, MCE values from pyruvate were plotted against MCE values from glutamate to assess which systemic substrate mitochondria in these samples prefer (Figure 8). Similarly, ADP-activated state 3 respiration based on either pyruvate or glutamate were plotted to visualize respiratory substrate preferences in Luminal A and TNBC subgroups (Figure 9). healthy breast tissue collected from the same patients during the surgery, but notably, this tissue has very low activity for both of these properties and hence these numbers are not significant to this study and have not been included in the data.

**Table 1:** Mitochondrial citrate efflux in five major human breast cancer subtypes grouped on pyruvate or glutamate as exogenous substrate. MCE values in ng citrate/mg wet tumor weight^*^h.

	**Pyruvate**	**No of patients**	**Glutamate**	**No of patients**
Luminal A	85.1 (±10.9)	19	46.5 (±5.8)	21
Luminal B	98.3 (±10.9)	17	48.1 (±9.8)	18
HER2	130.0 (±46.7)	4	69.7 (±12.1)	6
Triple negative	138.9 (±18.6)	8	80.0 (±15.1)	8
Luminal B/HER2+	146.6 (±62.2)	3	93.8 (±32.6)	3

## Discussion

### Steady-State Markers and Citrate Levels in MCF-7 and MDA-MB-231 Cell Lines

We have shown previously, using permeabilization technique on human breast cancer samples, that ADP-activated respiration in TNBC tumors is significantly higher than that in Luminal A breast cancers. Surprisingly, the same study confirmed that in MDA-MB-231 (TNBC subtype) and MCF7 (Luminal A subtype) cell lines, exact opposite respiration ratio was evident (5). In continuation of this work, we wanted next to understand links between mitochondrial respiration and mitochondrial metabolism.

Mitochondrial content in the named cell lines was found to be well in line with the respiration data from our previous study (5). That is, both mitochondrial content and ADP-activated respiration (Figure 6B) in MCF-7 cells are 2.5–3 times higher than that in MDA-MB-231 cells. MDA-MB-231 cell line, however, is representing clinically most aggressive TNBC subtype and therefore we reasoned that the aggressive phenotype has to be expressed on metabolic level in the mitochondria as described before (6). We first looked at steady-state metabolite level in whole cell extracts using GC-MS. The results revealed that despite citrate levels in both cell lines are in line with mitochondrial content and citrate synthase activity, significant differences are present for other TCA metabolites in MDA-MB-231 cells (Figure 3). Surprisingly, the differences were showing that the metabolite levels in MDA-MB-231, besides that for citrate, were higher than in MCF-7 cells. In support of the low citrate levels in MDA-MB-231 cells, citrate synthase expression and citrate synthase activity were both measured to be lower in MDA-MB-231 cells (Figure 2). In additional support for citrate level in MDA-MB-231 cell line, it has been previously shown that more aggressive tumors present with lower steady state levels of citrate (36, 37) and our findings confirm that understanding. We thereafter asked, that how can low citrate level justify central role of this metabolite in numerous cellular function that result in cancer cell homeostasis? For example, mitochondrial citrate, made available to cytosol via truncated TCA or reductive carboxylation from glutamate, feeds many cytosolic processes necessary for cell maintenance, survival and progression (11, 34, 35). Even more, high expression of citrate-malate antiporter that conducts MCE, has been associated with poor outcomes in both lung cancer and estrogen receptor negative breast cancers (38) and some authors have suggested this transporter as potential therapeutic target in cancers (39). Therefore, low steady-state citrate level in the more aggressive MDA-MB-231 cell line, despite expected by the literature, did not follow the importance of citrate in cancers. Possibly, the low citrate level is descriptive of high turnover of this metabolite pool, but steady-state methods are not sufficient in revealing this process.

### MCE and Glutamine Dependence in MCF-7 and MDA-MB-231 Cell Lines

In attempt to understand MCE activity on mitochondrial outer membrane, we developed a simple method as described in the Results section. Studies on MCE dynamics have been done previously, but only on (cancer) cell lines or on isolated mitochondria from soft physiological tissues like the liver (31). In the framework of this study, we tried to isolate mitochondria from human breast cancer samples, but it proved not possible due to rubbery texture of cancers. Similarly, we did not succeed in isolating mitochondria from physiological breast tissue (data not shown). Other methods have been suggested possibly solving this target. For example (40) used selective permeabilization and isotope tracing on cell monolayer, which, unfortunately, is not applicable on solid tumors. Rapid fractionation combined with isotope tracing, computational deconvolution and network modeling have been applied, but its again only suitable on cell lines (14). Additionally, respiration has been studied using homogenization of muscle tissue (17), but this destroys mitochondrial structure, removes regulatory effect of the cytoskeleton (22) and does not allow measurement of flux across mitochondrial membrane. Similarly, using labeled isotopes on human patients (4) is difficult and reaching reasonable patient selection across all breast cancer molecular subtypes, is expensive, time consuming and, most importantly, it is not possible to differentiate mitochondrial or cytosolic citrate pools nor the activity between these two compartments.

We ran our newly developed method initially on MDA-MB-231 and MCF7 cell lines. To our surprise, despite ADP-activated respiration in MCF-7 cells is three times higher (Figure 6B), and MDA-MB-231 are expectedly more glycolytic cells, both cell lines conduct MCE in equal amounts if pyruvate is the main substrate. But strikingly, if pyruvate is switched to glutamate, then MCF-7 cells fail to use it in similar amounts for MCE as MDA-MB-231 cells do (Figure 6A). The results reveal that MDA-MB-231 cells are 3 times more efficient in incorporating glutamate into cellular processes. In our experiments glutamate was added directly to the permeabilized samples, but in cellular context glutamate is mainly derived from glutamine through activity of glutaminase (41). These results are supported by previous reports that MDA-MB-231 cells will not survive glutamine withdrawal while MCF7 cells are glutamine independent (42).

Our data shows that MCF7 cells require more pyruvate for mitochondrial processes than the MDA-MB-231 cells, as they use it for both high respiration and high mitochondrial citrate efflux (Figures 6A,B). Dual use of pyruvate in the MCF7 cells equally for both respiration (generation of ATP) and MCE, might causally explain the capacity of MCF7 cells to overcome glutamine withdrawal. However, if glutamine is removed from the MDA-MB-231 cells or glutaminase is inhibited, then MDA-MB-231 cells do not have sufficient capacity to maintain requirements for ATP only through glycolysis (as OXHOS as additional source is nearly missing) and maintain necessary level of MCE (as ~32% of the MCE was derived from glutamate). Similarly, in SF188 glioblastoma cells 25% of fatty acyl carbons were derived from glutamine via MCE and the same for glucose was 60%, but complicated isotope based methods were applied to reach that conclusion (8).

Quantification of MCE in breast cancer cell lines confirmed that mitochondria are metabolic hubs for malignant cells and low/high respiration capacity does not lead to equally low/high metabolic activity. Importantly, mitochondrial amount or citrate synthase activity or citrate synthase expression do not predict metabolic activity in the given cell line. In conclusion, it can be predicted that TCA cycle and OXPHOS system are not directly coupled in all cell lines, however, both are necessary for cell survival and need to be simultaneously measured in order to sufficiently describe mitochondrial function.

### ADP-Activated Respiration and MCE in Human Breast Cancer Samples

Respiration in correctly permeabilized solid tumors can easily be jumpstarted with exogenous ADP if either pyruvate or glutamate are introduced to the test environment as substrates. From Figure 9 it can be easily appreciated that not all breast cancers display similar respiration patterns even within the same molecular subgroup. Among Luminal A diseases, some cancers tend to respire predominantly on pyruvate and only minimally on glutamate (e.g., lower left Luminal A patient marked as “A,” Figure 9), while some TNBC tumors conduct OXPHOS on both pyruvate or glutamate at very high level (TNBC patient marked with “B”) or rely mainly on glutamate for respiration (TNBC patient marked “C”). While Luminal A tumors seem to form a cluster on Figure 9, TNBC cancers display wide distribution in results which is in line with high heterogeneity in this subgroup (43).

As we showed above for cell lines, then also in solid human tumors respiration alone is presumably not sufficient for describing mitochondrial activity. Based on this knowledge, MCE rates were measured for all breast cancer samples. In Table 1, MCE rates are grouped based on breast cancer molecular subtypes (44) and metabolic substrate. On average, breast cancers tend to use less glutamate for MCE than pyruvate (similarly to cell lines), however, conversion rates from both metabolic substrates rise along expected aggressiveness of the tumors. Luminal A cancers are considered to have the most favorable outcome and TNBC cancers tend to be the most difficult to treat, and as our results clearly show, differences in MCE between these two opposing subtypes are significant for both pyruvate and glutamate as substrates (Figure 7). Therefore, despite high heterogeneity between human breast cancers, in metabolic terms, the differences for these two subtypes are clearly present and fall in line with expected aggressiveness of either subtype. For three patients diagnosed as being both luminal B and HER2+ the MCE rates were the highest. Even though this scarcely studied subtype is considered clinically aggressive (45), the number of patients in this subgroup was too low for making conclusions. For wider view across all cancer subtypes in this study, MCE values from pyruvate vs. glutamate were plotted and the wide distribution of metabolic preferences become clearly evident (Figure 8).

Together with our previous finding on respiration in clinical samples (5), it can be concluded that in contrary to cell lines, human breast tumors sustain ability to conduct OXPHOS and simultaneously direct citrate from TCA to feed anabolic processes in the cytosol. Both of these processes are synchronously increasing together with rising aggressiveness of the tumors. Importantly, results found above for two widely used cell lines (MCF7 and MDA-MB-231) proved to be the opposite in clinical samples. This finding can contribute to explanation why preclinical research often fails to translate to clinical phase as preclinical models do not sufficiently represent the human disease biology, especially in metabolic terms.

### Predictive Value of MCE and ADP-Activated Respiration in Clinical Samples

Given that inhibitors of glutaminolysis have reached clinical trials in TNBCs (46) and our results showed significant differences in glutamate use in cell lines, we asked if the combined data for MCE and state 3 respiration might be predictive for estimating treatment susceptibility in our clinical samples. Table 2 presents data for 6 TNBC patients included to this study. It is apparent that TNBC #4 is using both pyruvate and glutamate equally low for MCE, while the tumor has high preference for glutamate in conducting OXPHOS. Therefore, it can be predicted that the given tumor might more likely respond to glutaminase inhibition as it does not have higher capacity to use pyruvate for MCE or OXPHOS to compensate lost availability of glutamine derived glutamate. The same can be predicted for TNBC #3. However, TNBC #1 and TNBC #2 present with phenotype that can easily compensate loss of glutamate via high usage of pyruvate for both MCE and respiration. The data in Table 2 brings out ambiguity in some patients as evident with TNBC #5. TNBC#5 has high dependence on glutamate for conducting OXPHOS, however, the same tumor has only minimal use of glutamate for MCE. It can be predicted that loss of glutamine derived glutamate in the given tumor can easily be compensated by high use of pyruvate for MCE, but the tumors' ability to sustain OXPHOS and cell survival on pyruvate at sufficient level, is unknown. Similarly, TNBC #6 has by far the highest dependence on glutamate for MCE and also highest respiration rate on glutamate, however the role of high use of pyruvate for respiration in overcoming possible glutamate restriction, remains unknown. Although the present study can suggest possible causality for glutaminase inhibition success or failure in certain breast cancers, without further preclinical research or clinical trials, these suggestions remain hypothetical. However, the way we propose to analyse TNBC susceptibility for glutaminase inhibition is strongly supported by a very recent finding. The authors of the study describe a new compound for blocking glutamine availability in malignancies and conclude that glycolysis, OXPHOS and glutaminolysis in cancers are highly interdependent and lack noticeable plasticity (47). Therefore, if capacity for each three properties are separately known, then predicting treatment susceptibility becomes possible. Here we have done those measurements on human breast cancers where MCE in cytosol-free samples acts as a quantified marker for capacity of using glucose or glutamine while OXPHOS on the same substrates is also measured. As mentioned above, further studies are needed to confirm this hypothesis.

**Table 2:** Mitochondrial citrate efflux and state 3 respiration in human TNBC samples grouped on pyruvate or glutamate as exogenous substrates.

	**Mitochondrial citrate efflux**		**State 3 respiration**	
	**Pyruvate**	**Glutamate**	**Pyruvate**	**Glutamate**
TNBC #1	193.2	74.7	0.71	0.49
TNBC #2	144.6	83.3	1.30	0.67
TNBC #3	92.9	74.5	0.26	0.55
TNBC #4	69.6	65.1	0.57	1.08
TNBC #5	121.3	13.6	0.37	0.54
TNBC #6	94.5	161.7	1.05	2.02

MCE measured as ng citrate/mg wet tumor weight*h; state 3 respiration measured as nmol O_2_/min per mg wet tumor weight.

Additionally, as OXPHOS and MCE must be separately analyzed for both pyruvate and glutamate, then it can be concluded that segregating patients for anti-glutaminase treatment using only glutamine-based positron emission tomography (48) is not sufficient. This approach describes affinity for only one major substrate, while misses to describe cancers ability to compensate glutamine withdrawal with pyruvate-based MCE and/or OXPHOS (as shown above for TNBC #3, #4, #5, #6; Table 2).

### Limitations

This study includes a number of limitations. Firstly, it has not been detected if MCE from glutamate is coming through canonical TCA or reductively, nor were other mitochondrial metabolite effluxes included in the results besides citrate. Therefore, future studies with isotope tracing (including on solid tumors using the technique described here) are warranted. BTC can slightly affect respiration and it might affect the comparability of two parallels that we ran on all tumor samples and, additionally, it would be beneficial to design the analysis to work in a single chamber. Finally, it would have contributed to this study if a selection of patient samples would have been firstly analyzed for steady state metabolite levels (using rapid freezing of part the sample in the operating room) and later, for temporal MCE as described here.

## Conclusions

We have shown that permeabilization-based approach is rapid for segregating metabolic and respiratory profiles in clear and quantified manner for both cell lines and solid human tumors. The method presented here offers time dependent view of intracellular metabolism and does not require changes in patient management before or during the surgery nor is it that time-critical as described before for isotope-based methods.

We showed that scarcely respiring MDA-MB-231 cells present with higher MCE than highly respiring MCF-7 cells do and, therefore, it can be concluded that OXPHOS and TCA are not directly coupled in all cell lines. Our data proved that in terms of MCE and OXPHOS, these two widely used cell lines (MCF7, MDA-MB-231) do not replicate the same properties in respective human breast cancer samples. Surprisingly, the findings for the two cell lines and clinical samples were directly opposing and that can explain difficulties in translational research. In contrast to cell lines, capacity for OXPHOS and MCE in human breast tumors are increasing together with increasing clinical aggressiveness of the tumors. Interestingly, when we analyzed our detailed data on the clinical samples of the most aggressive TNBC subtype, possible predictive value for glutaminase inhibitor treatment emerged that need to be confirmed by additional trials. Taken together, new and easier methods that quantify time dependent metabolism in solid human cancers can bring out shortcomings in our model systems and direct translational research in delivering new treatments to patients faster.

## Data Availability Statement

The raw data supporting the conclusions of this article will be made available by the authors, without undue reservation, to any qualified researcher.

## Ethics Statement

The studies involving human participants were reviewed and approved by Tallinn Medical Research Ethics Committee. The patients/participants provided their written informed consent to participate in this study.

## Author Contributions

AK designed the study, developed temporal MCE quantification method, conducted most of the experiments, and wrote the manuscript. NT conducted the respiration studies together with quantification of citrate. LT and VC grew the cultured cells for experiments. SG developed the metabolite analysis method on GC-MS. IS, KL, LM, RK, and VV provided the fresh human samples for the experiments together with respective medical data. TK supervised the study and reviewed the manuscript. All authors contributed to the article and approved the submitted version.

## Conflict of Interest

AK is scientific founder and holds equity in a start-up company, Mitogro, which is active in developing predictive tools for cancer metabolism. AK and TK are inventors for pending patent application No. US15/651,003 covering use of the described method in predictive setting in oncology. The remaining authors declare that the research was conducted in the absence of any commercial or financial relationships that could be construed as a potential conflict of interest.

## Abbreviations

BTC: 1,2,3-benzene tricarboxylic acid
OXPHOS: oxidative phosphorylation
TNBC: triple negative breast cancer
MCE: mitochondrial citrate efflux
TCA: tricarboxylic acid cycle.
